# Analyzing childhood (0–59 months) malnutrition determinants in five West African countries of Gabon, Gambia, Liberia, Mauritania, and Nigeria using a generalized additive mixed model from DHS data

**DOI:** 10.3389/fnut.2025.1653772

**Published:** 2025-12-11

**Authors:** Reshav Beni, Shaun Ramroop, Faustin Habyarimana

**Affiliations:** School of Mathematics, Statistics and Computer Science, University of KwaZulu-Natal, Pietermaritzburg, South Africa

**Keywords:** malnutrition, composite index of anthropometric failure, generalized additive mixed models, West Africa, public health

## Abstract

**Background:**

Malnutrition remains a serious public health challenge across West Africa, contributing to high rates of illness and mortality among children. Despite ongoing efforts, its prevalence remains high, underscoring the need to better understand the factors driving poor nutritional outcomes.

**Methods:**

This study analyzed data from Demographic and Health Surveys (DHS) conducted in Gabon, Gambia, Liberia, Mauritania, and Nigeria. Advanced statistical models were used to explore how child, household, and environmental characteristics relate to malnutrition, measured using the Composite Index of Anthropometric Failure (CIAF). Key variables included child age and sex, recent illness, feeding practices, and access to clean water and cooking fuels.

**Results:**

Children aged 12–60 months had more than twice the odds of being malnourished compared to infants. Male children were more vulnerable than females. Protective factors included access to bottled or sachet water and clean cooking fuels, while recent fever and certain feeding practices—such as formula and fortified food consumption—were associated with increased risk. The analysis also revealed age-related patterns in malnutrition risk, with vulnerability peaking in early toddlerhood.

**Conclusion:**

This study highlights the complex interplay of health, environmental, and behavioral factors in child malnutrition across West Africa. The findings can guide evidence-based interventions focused on improving water and energy access, promoting dietary diversity, and integrating nutrition with disease prevention strategies to reduce malnutrition and improve child health outcomes.

## 1 Introduction

The World Health Organization (WHO) describes malnutrition as “deficiencies, excesses, or imbalances in a person’s intake of energy and/or nutrients,” which can lead to significant health consequences (1). This condition encompasses both undernutrition, which includes stunting, wasting, and micronutrient deficiencies, and overnutrition, such as obesity, both of which contribute to the growing burden of diet-related non-communicable diseases. Undernutrition remains a pressing global health concern, particularly in low- and middle-income countries. For instance, the WHO estimates that in 2016, approximately 155 million children under 5 years of age were affected by stunting, with undernutrition implicated in nearly 45% of deaths among this vulnerable population (2). The issue is especially acute in West Africa, where malnutrition is influenced by a multifaceted array of factors, including individual, social, economic, political, and environmental determinants (3). Addressing this complex challenge requires a comprehensive understanding of its underlying causes and their interactions.

Anthropometric indicators, such as mid-upper arm circumference (MUAC), weight-for-age, height-for-age, and weight-for-height, are widely utilized to assess undernutrition, which manifests as stunting, wasting, underweight, or micronutrient deficiencies. Wasting, identified by a low weight-for-height z-score, is indicative of acute malnutrition and is often associated with recent food insecurity, illness, or inadequate dietary intake. Stunting, characterized by a low height-for-age z-score, reflects chronic undernutrition and is frequently linked to prolonged factors such as poverty, poor maternal health, and suboptimal neonatal care. Underweight, determined by a low weight-for-age z-score, may suggest a combination of both stunting and wasting (4). Additionally, micronutrient deficiencies, which affect more than 2 billion people worldwide, involve insufficient intake or absorption of essential vitamins and minerals, including vitamin A, iron, iodine, and zinc. These deficiencies are often interconnected and can contribute to a vicious cycle of poor health, particularly in vulnerable populations. This cycle is perpetuated by maternal undernutrition during pregnancy and lactation, compounded by socioeconomic challenges such as poverty, limited access to healthcare, and food insecurity (5).

The severity of undernutrition is categorized into moderate acute malnutrition (MAM) and severe acute malnutrition (SAM). MAM is typically identified by a mid-upper arm circumference (MUAC) measurement between 115 mm and <125 mm, while SAM is diagnosed when MUAC falls below 115 mm or when nutritional edema is present (6). AM is especially critical, as it is associated with a tenfold increase in mortality risk compared to children with a z-score ≥ − 1. This heightened risk underscores the urgent need for effective interventions to address acute malnutrition, particularly in regions like West Africa, where its prevalence and impact are disproportionately high. Targeted strategies to mitigate the burden of malnutrition are essential, as the consequences of SAM extend beyond immediate health outcomes, affecting long-term growth, development, and socioeconomic stability (7).

Previous research by Salm et al. (3), Duru et al. (8), Igbokwe et al. (9), Oyekale (10), Sawadogo et al. (11), and Uthman and Aremu, (12) has primarily focused on individual conventional anthropometric indices, such as stunting, underweight, and wasting, as outlined by the World Health Organization (WHO) (4). While these indices offer critical insights into specific dimensions of malnutrition, their isolated use often fails to provide a comprehensive understanding of childhood malnutrition. This limitation stems from the fact that each index measures a distinct aspect of nutritional status, potentially neglecting children who experience overlapping or concurrent forms of malnutrition (13). To bridge this gap, the composite index of anthropometric failure (CIAF) has been introduced as a more holistic approach. The CIAF integrates multiple forms of malnutrition into a single measure, enabling a more accurate and nuanced assessment of the overall burden of childhood malnutrition. By capturing the interplay between different malnutrition indicators, the CIAF helps identify children who may be misclassified or overlooked when relying solely on traditional, isolated indices (13, 14). This approach provides a more robust framework for understanding and addressing the multifaceted nature of malnutrition.

The selection of these countries was driven by the limited availability of existing research and the accessibility of up-to-date, high-quality data, which facilitate a timely and impactful analysis. While earlier studies, such as those by Duru et al. (8), and Igbokwe et al., (9) primarily examined sociodemographic determinants of malnutrition, this study expands the analytical framework to incorporate a wider range of factors. These include dietary variables (e.g., consumption of milk, formula feeds, tubers, eggs, and meat), sanitation indicators (access to clean water and toilet facilities), clinical factors (e.g., anemia, fever, and cough).

To capture the complex, non-linear relationships between these variables and malnutrition outcomes, this study employs a Generalized Additive Mixed Model (GAMM), a robust statistical approach that allows for flexible modeling of fixed and random effects while accounting for spatial and temporal heterogeneity (15, 16). By integrating these diverse dimensions and leveraging advanced analytical techniques, this study offers a more holistic perspective on the drivers of malnutrition, marking a significant advancement over previous research. This comprehensive approach not only enhances the understanding of the complex and interrelated factors contributing to malnutrition but also provides valuable insights for designing targeted and effective intervention strategies in Sub-Saharan Africa (17). The inclusion of these multifaceted variables and innovative methodologies underscores the novelty of this study and its potential to inform policies aimed at addressing malnutrition in the region.

## 2 Methodology

### 2.1 Data

This study is based on a secondary analysis of data from the Demographic and Health Surveys (DHS) conducted in West African countries. The DHS employs a complex, two-stage sampling design that incorporates stratification and clustering, with unequal selection probabilities to ensure the sample is nationally representative. Access to the data was obtained through a formal application process, which was submitted to and approved by the DHS program.

In line with DHS protocols, the data were extracted from the Kids Recode table, which includes information on children under 5 years of age born to women interviewed in the surveys. This table provided all the necessary variables for the analysis, eliminating the need for additional data manipulation or table creation. A consolidated dataset was constructed by combining Kids Recode tables from surveys conducted in Gabon (2019–2021), Gambia (2019–2020), Liberia (2019–2020), Mauritania (2019–2021), and Nigeria (2021). To ensure the representativeness of the sample and address disparities in sampling proportions, population adjustment weights were applied to account for variations in cluster selection probabilities. Missing data points were excluded from the study.

### 2.2 Independent variables

This study investigated demographic, socioeconomic, and environmental factors that have been consistently linked to child malnutrition in prior research. These factors guided the selection of independent variables, which encompassed: the mother’s current age (treated as a continuous variable), type of residence, maternal education level, source of drinking water, type of toilet facility, access to electricity, gender and age of the household head (treated as a continuous variable), type of cooking fuel, household wealth, and the use of mosquito bed nets. Additionally, child-specific variables were included, such as dietary practices (e.g., consumption of milk, formula, fortified foods, tubers, eggs, or meat), anemia levels, health facility visits, child weight, child age (treated as a continuous variable) and recent illnesses like fever or cough. After evaluating potential interaction terms within the Generalized Additive Mixed Model (GAMM), no statistically significant interactions were found.

The theoretical foundation of this study is grounded in established literature, including contributions by Duru et al. (8), Igbokwe et al. (9), Oyekale (10), Sawadogo et al. (11), Habyarimana et al. (18), Habyarimana, et al. (19). This framework synthesizes well-supported theories and concepts to guide the selection of variables hypothesized to influence malnutrition. It offers a systematic approach for examining the intricate relationships among demographic, socioeconomic, and environmental factors that contribute to child malnutrition in the study region. By building on these foundational works, the study aims to provide a comprehensive understanding of the multifaceted drivers of malnutrition.

### 2.3 Dependent variable

The Composite Index of Anthropometric Failure (CIAF), developed by Svedberg (20), provides a holistic framework for evaluating nutritional status by consolidating multiple dimensions of malnutrition into a unified metric. This innovative approach overcomes a significant limitation of conventional indices—such as stunting, underweight, and wasting—which are often applied in isolation and may inadequately represent the full spectrum of malnutrition due to their failure to account for the co-occurrence of undernutrition conditions (20). Traditional reliance on single indices can underestimate the true prevalence of anthropometric failure within populations, as these methods do not adequately capture the overlapping and concurrent manifestations of malnutrition (21). The CIAF thus offers a more nuanced and comprehensive tool for assessing nutritional disparities and guiding targeted interventions.

The CIAF, as outlined in the *“Handbook of Anthropometry: Physical Measures of Human Form in Health and Disease,”* integrates three key anthropometric indicators—weight-for-age (WAZ), height-for-age (HAZ), and weight-for-height (WHZ)—to assess the nutritional status of children under 5 years of age. This comprehensive framework classifies nutritional status into distinct categories: (A) no failure (normal); (B) wasting only; (C) wasting and underweight; (D) wasting, underweight, and stunting; (E) underweight and stunting; (F) stunting only; and (Y) underweight only (22).

By consolidating these categories, the CIAF offers a comprehensive perspective on anthropometric failure, encompassing the full spectrum of undernutrition. This integrated approach enables a more precise estimation of the malnutrition burden within a population by identifying children experiencing multiple nutritional deficiencies concurrently. The overall anthropometric failure rate is calculated as the sum of categories B, C, D, E, F, and Y, which collectively capture all manifestations of undernutrition. This method ensures a more accurate and inclusive assessment of nutritional disparities, highlighting the complexity and overlap of malnutrition conditions (20, 21). Table 1 provides a detailed description of the various categories of CIAF (14).

**Table 1:** Category anthropometric failure in under 5 year using CIAF.

Group	CIAF categories	Description	Wasting	Stunting	Underweight
A	No failure	Children whose height and weight are above the age-specific norm and who do not suffer from any anthropometric failure	No	No	No
B	Wasting only	Children with acceptable weight and height for their age, but who have subnormal weight for height	Yes	No	No
C	Wasting and underweight	Children with above-norm heights, but whose weight for age and weight for height are too low	Yes	No	Yes
D	Wasting, underweight and stunting	Children who suffer from anthropometric failure on all three measures	Yes	Yes	Yes
E	Stunting and underweight	Children with low weight for age and low height for age, but who have acceptable weight for their height	No	Yes	Yes
F	Stunting only	Children with low height for age but who have acceptable weight, both for their age and for their short height	No	Yes	No
Y	Underweight only	Children who are only underweight – whose weight for age is acceptable	No	No	Yes

In conclusion, the Composite Index of Anthropometric Failure (CIAF) serves as a powerful tool for identifying diverse forms of anthropometric failure, providing a detailed and holistic measure that deepens our understanding of childhood malnutrition. By capturing the multifaceted nature of nutritional deficiencies, the CIAF enables the development of more targeted and effective intervention strategies to address the complex challenges of undernutrition (23).

**Figure 1:**
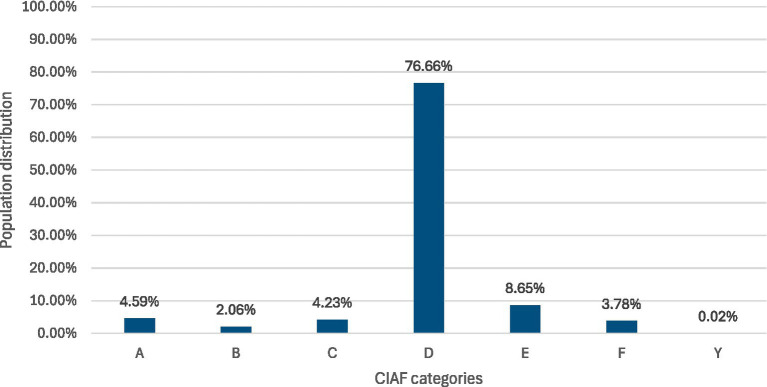
Overall distribution of CIAF categories of children (0-59 m) in Gabon (2019–2021), Gambia (2019–2020), Liberia (2019–2020), Mauritania (2019–2021), and Nigeria (2021).

**Figure 2:**
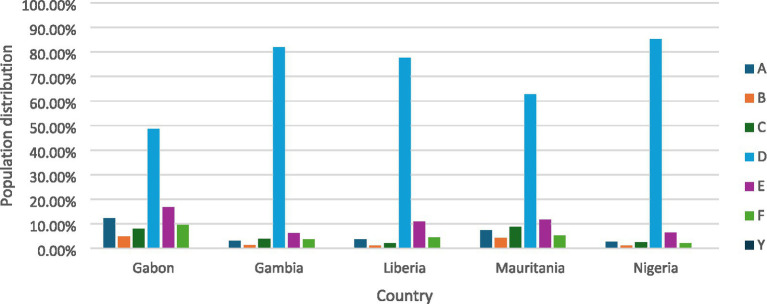
Country level distribution of CIAF categories of children (0-59 m) in Gabon (2019–2021), Gambia (2019–2020), Liberia (2019–2020), Mauritania (2019–2021), and Nigeria (2021).

Figures 1, 2 display the distribution of the outcome for the pooled and country level data sample.

## 3 Statistical analysis

Generalized Additive Mixed Models (GAMMs) are an extension of Generalized Additive Models (GAMs) that incorporate both fixed and random effects, making them particularly suited for analyzing complex datasets with nonlinear relationships, hierarchical structures, or temporal/spatial dependencies (15, 16). GAMMs allow for the flexible modeling of nonlinear predictor effects using smooth functions for continuous variables, while also accommodating categorical variables as fixed effects. This makes them ideal for applications in ecology, epidemiology, and social sciences, where data often exhibit a mixture of continuous and categorical predictors (24).

In this study, we employed GAMMs to model the relationships between a combination of continuous and categorical predictors and the response variable. The analysis was conducted using PROC GAMPL in SAS, a powerful procedure for fitting GAMMs using penalized likelihood estimation (25). PROC GAMPL is particularly well-suited for large datasets and complex models, as it uses efficient algorithms to estimate smooth functions and random effects. Statistical significance of independent variables was determined at the *p* < 0.05 level.

The GAMM was specified as follows:


$$\begin{array}{l} g(E(Y_{\mathrm{ij}}))=\beta_{0}+f_{1}(x_{1\mathrm{ij}})+f2(x_{2\mathrm{ij}})+\ldots +f_{p}(x_{\mathrm{pij}})\\ +\gamma_{1}z_{1\mathrm{ij}}+\gamma_{2}z_{2\mathrm{ij}}+\ldots +\gamma_{p}z_{\mathrm{qij}}+Z_{\mathrm{ij}}b_{i}+e_{\mathrm{ij}} \end{array}$$

Where:

$Y_{\mathrm{ij}}$ is the response variable for the $j^{\mathrm{th}}$ observation in the $i^{\mathrm{th}}$ group.

g(.) is the link function.

$\beta_{0}$ is the intercept.

$f_{1},f_{2},\ldots ,f_{p}$ are smooth functions for the nonlinear effects of continuous predictors $x_{1},x_{2},\ldots ,x_{p.}$

$z_{1},z_{2},\ldots ,z_{q}$ are categorical predictors with fixed effects $\gamma_{1},\gamma_{2},\ldots ,\gamma_{q}$

$Z_{\mathrm{ij}}$ is the design matrix for random effects

$b_{i}$ represents the random effects for the $i^{\mathrm{th}}$ group, assumed to follow a normal distribution $b_{i}\sim N(0,\Sigma )$

$e_{\mathrm{ij}}$ is the residual error term

Smooth functions $f_{k}$ for continuous predictors were modeled using penalized regression splines, with the degree of smoothness determined by generalized cross-validation (GCV) (26).

A Generalized Additive Mixed Model (GAMM) was employed to investigate the predictors of child anthropometric failure, as measured by the Composite Index of Anthropometric Failure (CIAF). This modeling framework enabled the estimation of non-linear relationships between continuous covariates and malnutrition, while also accounting for potential clustering effects at the household or community level.

To implement the GAMM, the study utilized PROC GAMPL in SAS, which allowed for flexible modeling of both continuous and categorical predictors. Smooth functions were applied to continuous variables such as the current age of the mother, child’s weight, and age of the household head. Categorical predictors included maternal education level, household water source, toilet facility type, electricity access, gender of the household head, cooking fuel type, household wealth index, child feeding practices, health service utilization, anemia level, and recent illness symptoms. Random effects were incorporated to appropriately model the hierarchical structure of the data and address clustering at the household or community level.

## 4 Results

The results outlined in Table 2 revealed several significant covariates associated with the likelihood of a child being classified as malnourished. Access to improved water sources and cleaner cooking fuels was associated with lower odds of malnutrition. Specifically, children in households that used bottled, or sachet water had 67% lower odds of CIAF (OR = 0.33, *p* < 0.0001) relative to those using piped water. Similarly, gas cooking fuel use was associated with significantly reduced odds of malnutrition (OR = 0.52, *p* < 0.0001).

**Table 2:** Results from the GAMM model.

Variable name	Estimate	Odds ratio	*p*-value
Residence type (Ref = Urban)			
Rural	−0.098633	0.9061	0.1285
Mother’s highest education level (Ref = Secondary)			
Primary	0.17354	1.1895	0.0109
No education	0.161088	1.1748	0.0172
Higher	−0.068965	0.9334	0.4221
Source of drinking water (Ref = Piped)			
Bottle/Sachet	−1.109403	0.3298	<0.0001
Natural Sources	−0.22274	0.8003	0.015
Tanker	−0.00619	0.9938	0.9651
Well	−0.354962	0.7012	<0.0001
Other	0.18609	1.2045	0.1326
Type of toilet facility (Ref = Flush)			
Pit	−0.205081	0.8146	0.0025
Other	−0.088866	0.915	0.2176
Household has electricity (Ref = Yes)			
No	−0.030993	0.9695	0.67
Gender of the head of the household (Ref = Male)			
Female	−0.084468	0.919	0.1505
Type of cooking fuel (Ref = Wood)			
Electricity	−0.163535	0.8491	0.4898
Gas	−0.663166	0.5152	<0.0001
No Food cooked	−1.244809	0.288	0.1205
Wealth Index (Ref = Richest)			
Poorest	−0.016215	0.9839	0.8821
Poorer	0.094093	1.0987	0.3514
Middle	0.148745	1.1604	0.0888
Richer	0.07471	1.0776	0.3205
Slept under a net last night (Ref = No net)			
Only treated nets	0.273281	1.3143	0.0139
Only untreated nets	0.30508	1.3567	0.0046
Both treated and untreated	1.034006	2.8123	0.4148
Visted a health facility in the last 12 months (Ref = Yes)			
No	−0.077502	0.9254	0.1311
Gave the child milk (Ref = No)			
Yes	−0.066752	0.9354	0.2825
Gave the child formula (Ref = No)			
Yes	0.23725	1.2678	0.0004
Gave the child fortified food (Ref = No)			
Yes	0.317546	1.3738	<0.0001
Gave the child tubers (Ref = No)			
Yes	−0.046413	0.9546	0.5235
Gave the child eggs (Ref = No)			
Yes	0.066485	1.0687	0.4145
Gave the child meat (Ref = No)			
Yes	0.072615	1.0753	0.3564
Has a mosquito net (Ref = Yes)			
No	−0.248154	0.7802	0.0002
Gender of the child (Ref = Male)			
Female	−0.265084	0.7671	<0.0001
Fever in the last 2 weeks (Ref = No)			
Yes	−0.231937	0.793	0.0008
Cough in the last 2 weeks (Ref = No)			
Yes	0.118304	1.1256	0.0778
Anemia (Ref = Not Anemic)			
Anemic	0.207403	1.2305	0.0002
Child weight (Ref = Greater than 1.5 kg)			
Child_weight_group 1. Less than 500 g	−0.670371	0.5115	0.024
Child_weight_group 2. Between 500 g and 1 kg	−1.85586	0.1563	<0.0001
Child_weight_group 3. Between 1 kg and 1.5 kg	−1.664605	0.1893	<0.0001
Age of household head (Ref = Greater than 65 years)			
1.less than 18 years	2.044702	7.7269	0.0549
2.less than 25 years	0.183687	1.2016	0.2027
3.less than 35 years	0.352966	1.4233	0.0003
4.less than 45 years	0.171406	1.187	0.0681
5.less than 55 years	0.090435	1.0947	0.3604
6.less than 65 years	0.117184	1.1243	0.2651

Gender differences were also observed. Female children had significantly lower odds of anthropometric failure compared to males (OR = 0.77, *p* < 0.0001). This finding is in line with the biological literature indicating that male infants may be more susceptible to morbidity and growth faltering during early life (27). The presence of fever in the 2 weeks preceding the survey was positively associated with CIAF (OR = 1.26, *p* = 0.0008).

With respect to dietary practices, consumption of infant formula (OR = 1.27, *p* = 0.0004) and fortified foods (OR = 1.37, *p* < 0.0001) was unexpectedly associated with increased odds of malnutrition. These associations may reflect reverse causality or residual confounding—for example, mothers may turn to formula or fortified products in response to growth faltering rather than as preventive practices. This complexity underlines the need for further qualitative and longitudinal investigation into feeding behaviors.

Children from households with lower maternal education, particularly those where the mother had only completed primary education (OR = 1.19, *p* = 0.0109) and had no education (OR = 1.17, *p* = 0.0172), were at greater risk of malnutrition. Additionally, the age of the household head appeared to matter: households headed by adults aged 25–35 years were associated with higher odds of child malnutrition (OR = 1.42, *p* = 0.0003), potentially reflecting differences in wealth accumulation, caregiving capacity, or generational caregiving dynamics.

The final GAMM model was selected based on penalized likelihood, Generalized Cross Validation (GCV), and the Bayesian Information Criterion (BIC), all of which supported the adequacy and parsimony of the fitted model. No statistically significant interactions were retained, indicating that the primary effects captured the major sources of variation in malnutrition risk.

**Figure 3:**
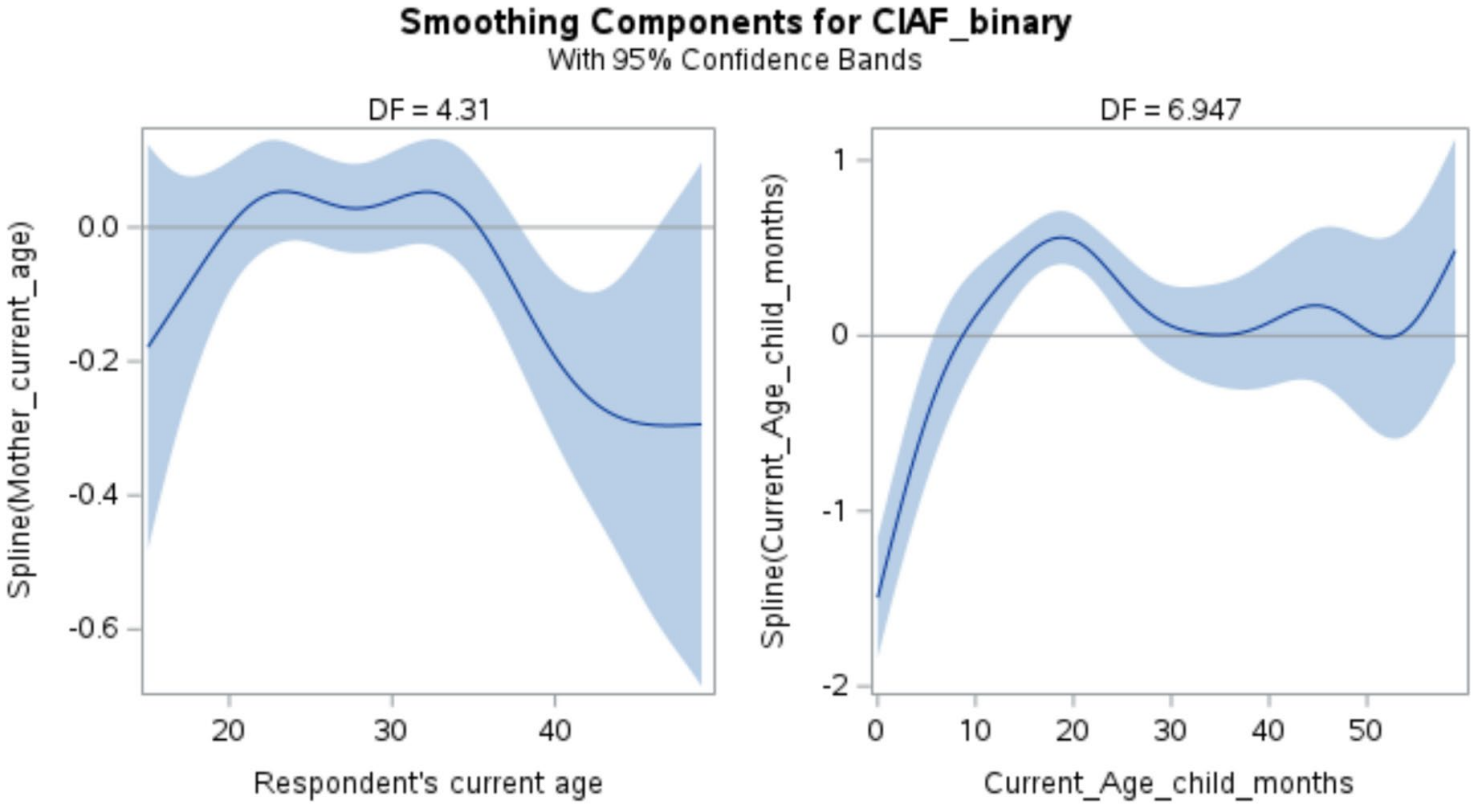
Smoothing components for the composite index of anthropometric failure.

Figure 3 and Table 3 illustrates the smoothing components for the Composite Index of Anthropometric Failure (CIAF) binary outcome, along with 95% confidence bands, providing insights into the relationships between various predictors and the likelihood of anthropometric failure in children.

**Table 3:** Continuous variable results.

Tests for smoothing components				
Component	DF	DF for Test	Chi-Square	Pr > ChiSq
Spline(Mother_current_age)	4.31024	6	13.3338	0.0380
Spline(Current_Age_child_months)	6.94699	8	459.2280	<0.0001

The smoothing terms for both maternal age (Mother_current_age) and child age in months (Current_Age_child_months) were statistically significant, indicating meaningful non-linear relationships with the binary malnutrition outcome. In this context, a non-linear relationship means that the effect of age on malnutrition does not follow a straight line — for example, the risk of malnutrition does not increase or decrease steadily as the mother or child gets older. Instead, the risk may go up at some ages and down at others. This modeling flexibility allows one to uncover realistic, data-driven patterns in malnutrition risk across age groups that a standard linear approach would overlook (16, 28).

The test for smoothing components (Table 3) revealed that the spline for maternal age was significant (*χ*^2^ = 13.33, df = 6, *p* = 0.038), with an effective degrees of freedom (EDF) of 4.31, suggesting moderate non-linearity. The spline for child age was highly significant (*χ*^2^ = 459.23, df = 8, *p* < 0.0001) and showed a more complex functional form (EDF = 6.95). Here, moderate non-linearity refers to a pattern that is more flexible than a straight line but not extremely variable. An EDF of around 4.3 implies that the relationship can bend in several places — for instance, to capture a rise, plateau, and fall — but without overfitting the data. In contrast, the EDF for child age (~6.95) suggests a more complex shape with multiple inflection points, capturing the nuanced variation in malnutrition risk as children develop across infancy, toddlerhood, and early childhood (16).

Figure 3 displays the estimated smoothing functions with 95% confidence intervals. The relationship between maternal age and malnutrition risk exhibited a U-shaped pattern, with higher predicted malnutrition risk observed for children of younger (<25 years) and older (>35 years) mothers, and lower risk in the intermediate age range (25–35 years).

For child age, the estimated effect showed lower malnutrition risk in early infancy (0–10 months), an increase in risk during late infancy and early toddlerhood (~10–20 months), followed by a decline and stabilization in risk through toddlerhood (~20–35 months). These non-linear patterns reflect known vulnerabilities during key developmental windows. For instance, the risk of malnutrition is high during infancy due to exclusive breastfeeding challenges, delayed initiation of complementary feeding, or poor dietary diversity (29–31).

As children transition into toddlerhood, their diets typically diversify and stabilize, contributing to improved nutritional outcomes. However, risk may rise again during preschool years due to factors such as reduced caregiver attention, exposure to infections, or household food insecurity (32, 33). These findings reinforce the need for continuous, age-specific nutrition support and health interventions, particularly in low-resource settings.

## 5 Discussion

Malnutrition continues to be a pervasive and deeply rooted issue in the West African nations of Gabon, Gambia, Liberia, Mauritania, and Nigeria, significantly contributing to high rates of childhood illness and mortality. Despite ongoing efforts by governments, nonprofit organizations, and humanitarian agencies to implement healthcare policies designed to improve access to essential services, the persistently high prevalence of malnutrition indicates that current strategies have fallen short.

Recent global assessments underscore this concern. According to the 2025 Joint Child Malnutrition Estimates by UNICEF, WHO, and the World Bank, progress in reducing child malnutrition has stalled in many regions. Just 28% of countries are currently on track to halve the number of children affected by stunting by 2030, and only 17% are on track to meet the target for reducing childhood overweight. Moreover, for over one-third of countries, progress toward the wasting target cannot be assessed due to data gaps. These findings highlight the urgent need for more proactive, targeted, and sustained interventions to address the root causes of malnutrition and accelerate progress toward global nutrition goals (34).

This study has identified several critical determinants of malnutrition that emerged, reflecting a complex interplay of demographic, environmental, and behavioral factors. Children’s age emerged as a key factor influencing malnutrition risk, with vulnerability increasing as they grow older, likely due to challenges associated with weaning, inadequate complementary feeding, and increased exposure to infections. The estimated effect showed lower malnutrition risk in early infancy (0–10 months), an increase in risk during late infancy and early toddlerhood (~10–20 months), followed by a decline and stabilization in risk through toddlerhood (~20–35 months). This pattern aligns with known nutritional transitions and developmental vulnerabilities during early childhood, reinforcing the need for targeted interventions during the weaning and early feeding stages. This elevated risk among older children is consistent with prior studies demonstrating a sharp decline in growth velocity after the first year of life, often attributable to inadequate complementary feeding, exposure to unhygienic environments, and increased risk of infection (29, 30, 33).

Access to improved water sources and clean cooking fuels was associated with lower malnutrition risk, underscoring the importance of household infrastructure in shaping child health outcomes, which is supported by research done by (35, 36).

Gender differences were also observed, with male children at greater risk, aligning with biological and possibly sociocultural susceptibilities. This finding is in line with the biological literature indicating that male infants may be more susceptible to morbidity and growth faltering during early life (27).

Maternal education played a protective role, emphasizing the importance of women’s empowerment and health literacy. Additionally, recent illness, particularly fever, was positively associated with malnutrition, reinforcing the well-established link between infection and undernutrition, reaffirming the well-documented bidirectional relationship between infection and undernutrition (37).

Dietary practices such as the use of infant formula and fortified foods showed associations with increased risk, suggesting the need for closer scrutiny of feeding patterns and possible underlying socioeconomic or health factors. These findings highlight the multifactorial nature of malnutrition and the need for integrated interventions that address both immediate and structural determinants.

Despite limited recent data on the determinants of malnutrition in Gabon, Gambia, Liberia, Mauritania, and Nigeria, this study’s findings align with and diverge from evidence in other regions. For instance, Fenta et al. (38), using Ethiopian Demographic and Health Surveys data and a generalized linear mixed model (GLMM), found that factors such as female gender, absence of comorbidities, and first birth order were associated with lower CIAF prevalence, consistent with this study’s finding that female children have a reduced risk of malnutrition. However, unlike this study, they identified rural residence and maternal underweight as significant predictors, which were not significant in our analysis. Similarly, Fenta et al. (39), highlighted maternal education and drinking water source as key determinants of CIAF using a spatial autocorrelation model, aligning with our finding that bottled/sachet water is protective against malnutrition. However, they did not find significant associations with household wealth or sanitation facilities, which contrasts with our results showing no significant link between toilet facility type and CIAF.

Sahiledengle and Mwanri (40), using multilevel mixed-effects negative binomial regression on the 2019 Ethiopia Mini Demographic and Health Survey, identified child gender and age as significant predictors of CIAF, consistent with our findings that female children and older children (12 to 60 months) are at higher risk. However, they did not find significant associations with toilet facility type, maternal education, or place of residence, which differs from our results. Similarly, Pomati and Nandy (41), analyzing malnutrition trends across West and Central Africa, observed associations with household wealth and rural residence, which were not significant in our study. Ayres et al. (42) using multilevel binary logistic regression on 2019 Ethiopian demographic data, also noted associations between CIAF and child gender, age, and maternal education, but did not find source of drinking water or toilet type to be significant, contrasting with our findings.

These studies collectively underscore regional variations in the determinants of CIAF while confirming several shared risk factors, such as child age and gender. However, the unique findings of this study—such as the protective effects of bottled/sachet water and gas for cooking, as well as the association between fever and increased malnutrition risk—highlight the importance of context-specific analyses to inform targeted interventions.

The findings of this study highlight the need for targeted interventions to address the significant determinants of malnutrition. Access to safe drinking water and clean cooking fuels emerged as critical protective factors, underscoring the importance of improving infrastructure in high-risk areas. Public health policies should prioritize expanding clean water distribution systems, promoting water purification technologies, and ensuring access to affordable, clean cooking fuels such as gas. These measures can reduce environmental risks and improve nutritional outcomes, particularly in underserved communities (43).

The age of the child and gender disparities in malnutrition risk call for age- and gender-sensitive interventions. Older children (12 to 60 months) are at significantly higher risk, suggesting the need for targeted nutritional support during this critical developmental period. Programs such as school feeding initiatives and community nutrition education should be strengthened to ensure adequate dietary intake for this age group. Additionally, the higher vulnerability of male children to malnutrition warrants further investigation into biological and sociocultural factors, as well as tailored interventions to address their specific needs (44).

The association between fever in the last 2 weeks and increased malnutrition risk highlights the interconnectedness of infectious diseases and malnutrition. Public health strategies should integrate malnutrition screening with infectious disease prevention programs, such as malaria control initiatives and the promotion of mosquito net use. Strengthening healthcare access to ensure early detection and treatment of both malnutrition and infectious diseases is essential to mitigate their combined impact on child health (45, 46). In this context, promoting Community Management of Acute Malnutrition (CMAM) and Simplified Protocols is particularly important. These approaches support the joint diagnosis and treatment of infectious diseases and malnutrition, while also decentralizing care through community health workers and streamlining treatment protocols to improve coverage and efficiency, especially in emergency and resource-limited settings (47, 48).

The finding that milk consumption is associated with increased odds of malnutrition is counterintuitive and requires further investigation. Public health programs should promote dietary diversity and balanced nutrition, ensuring that milk is complemented with other nutrient-dense foods. Community nutrition initiatives and school feeding programs can play a vital role in educating caregivers about optimal feeding practices and improving access to diverse, affordable foods (49, 50).

Finally, the strong association between child weight and malnutrition underscores the importance of routine growth monitoring and early intervention. Healthcare systems should prioritize regular screening for nutritional deficiencies and provide timely support to at-risk children. Integrating nutritional counseling and supplementation into maternal and child health services can help prevent malnutrition and its long-term consequences (44).

While this study provides important insights into the determinants of child malnutrition, further research is needed to explore additional contributing factors. These include household wealth indicators, family health history, and the severity of conditions such as anemia, tuberculosis, and HIV. Moreover, vaccination coverage, vitamin A supplementation, deworming practices, and adherence to chronic medications warrant closer examination for their potential roles in child health outcomes.

It is also essential to investigate the social dimensions of malnutrition, such as the involvement of primary caregivers, the presence of healthcare chaperones, and the criteria for accessing government economic grants. To explore these complex and interrelated factors, alternative methodologies—such as structural equation modeling (SEM) or machine learning approaches like random forests and gradient boosting—could be employed. These techniques are well-suited to capturing nonlinear relationships and interactions that traditional models may overlook.

Finally, longitudinal studies are needed to uncover long-term patterns and trends in malnutrition. Such studies would help overcome the limitations of cross-sectional data and provide a more comprehensive understanding of persistent health challenges in the region, thereby informing sustainable and evidence-based interventions.

## 6 Conclusion

This study applied Generalized Additive Mixed Models (GAMMs) to examine the determinants of child malnutrition in five West African countries using the Composite Index of Anthropometric Failure (CIAF). The findings reveal that malnutrition is influenced by a complex interplay of demographic, environmental, and health-related factors. Older children and male children were found to be more vulnerable, while access to safe drinking water and clean cooking fuels emerged as protective. Health indicators such as recent fever and low birth weight were positively associated with malnutrition, and dietary practices—particularly milk consumption—showed unexpected associations that warrant further attention.

These results underscore the need for integrated, context-specific interventions that address both immediate and structural drivers of malnutrition. By targeting key risk factors through coordinated public health strategies, stakeholders can make meaningful progress in improving child nutrition and reducing the burden of malnutrition across West Africa.

## Data Availability

The data analyzed in this study was obtained with permission from the DHS program: https://dhsprogram.com/data/available-datasets.cfm. Requests to access these datasets should directed to the DHS program.
